# Trends for Readmission and Mortality After Heart Failure Hospitalisation in Malaysia, 2007 to 2016

**DOI:** 10.5334/gh.1108

**Published:** 2022-03-08

**Authors:** Yvonne Mei Fong Lim, Su Miin Ong, Stefan Koudstaal, Wen Yea Hwong, Houng Bang Liew, Jeyamalar Rajadurai, Diederick E. Grobbee, Folkert W. Asselbergs, Sheamini Sivasampu, Ilonca Vaartjes

**Affiliations:** 1 Julius Center for Health Sciences and Primary Care, University Medical Center Utrecht, Utrecht University, Utrecht, the Netherlands; 2 Institute for Clinical Research, National Institutes of Health, Ministry of Health Malaysia, Selangor, Malaysia; 3 Department of Cardiology, Division of Heart and Lungs, University Medical Center Utrecht, Utrecht University, Utrecht, the Netherlands; 4 Dept of Cardiology, Groene Hart Ziekenhuis, Gouda, the Netherlands; 5 Department of Cardiology, Queen Elizabeth II Hospital, Ministry of Health Malaysia, Sabah, Malaysia; 6 Department of Cardiology, Subang Jaya Medical Centre, Selangor, Malaysia; 7 Julius Global Health, Julius Center for Health Sciences and Primary Care, University Medical Center Utrecht, Utrecht University, Utrecht, the Netherlands; 8 Julius Clinical, Zeist, the Netherlands; 9 Institute of Cardiovascular Science and Institute of Health Informatics, Faculty of Population Health Sciences, University College London, London, United Kingdom

**Keywords:** outcomes, heart failure, trends, ethnicity

## Abstract

**Background and objectives:**

Data on population-level outcomes after heart failure (HF) hospitalisation in Asia is sparse. This study aimed to estimate readmission and mortality after hospitalisation among HF patients and examine temporal variation by sex and ethnicity.

**Methods:**

Data for 105,399 patients who had incident HF hospitalisations from 2007 to 2016 were identified from a national discharge database and linked to death registration records. The outcomes assessed here were 30-day readmission, in-hospital, 30-day and one-year all-cause mortality.

**Results:**

Eighteen percent of patients (n = 16786) were readmitted within 30 days. Mortality rates were 5.3% (95% confidence interval (CI) 5.1–5.4%), 11.2% (11.0–11.4%) and 33.1% (32.9–33.4%) for in-hospital, 30-day and 1-year mortality after the index admission. Age, sex and ethnicity-adjusted 30-day readmissions increased by 2% per calendar year while in-hospital and 30-day mortality declined by 7% and 4% per year respectively. One-year mortality rates remained constant during the study period. Men were at higher risk of 30-day readmission (adjusted rate ratio (RR) 1.16, 1.13–1.20) and one-year mortality (RR 1.17, 1.15–1.19) than women. Ethnic differences in outcomes were evident. Readmission rates were equally high in Chinese and Indians relative to Malays whereas Others, which mainly comprised Indigenous groups, fared worst for in-hospital and 30-day mortality with RR 1.84 (1.64–2.07) and 1.3 (1.21–1.41) relative to Malays.

**Conclusions:**

Short-term survival was improving across sex and ethnic groups but prognosis at one year after incident HF hospitalisation remained poor. The steady increase in 30-day readmission rates deserves further investigation.

## Introduction

Heart failure (HF) is both a debilitating and costly clinical syndrome, with an expenditure of 346 billion US$ globally [1]. Despite medical advancement in the management acute coronary syndromes, the five-year survival rates for HF is poorer than most cancers [2]. Nevertheless, cardiac remodelling and progression to HF can be modified by appropriate preventive strategies and timely effective treatments are available. Estimates on the average prognosis of HF at the population level are important for monitoring changes in healthcare delivery. On the individual patient level, knowing the absolute risks of mortality and readmission enables shared decision-making between health providers and patients when making plans for disease management [3].

As it is in high-income countries, HF is already demonstrated to be a major burden in middle-income countries and this is expected to pose an equal, if not greater financial and morbidity impact as some of these countries are still grappling with a concurrent infectious disease burden [4]. The amount of data published on the epidemiology of HF from these countries is highly disproportionate compared to developed nations. Relying on data extrapolations from high-income countries is inadequate given the differences in healthcare infrastructure, health spending and demographic composition. The Asian HF registry, which included 11 countries in the region, found that one-year mortality rate was 9.6% among registry patients [5]. However, it was also noted that patients enrolled in this registry were mainly treated in academic hospitals with echocardiography expertise and more resources, and hence not necessarily representative of overall hospitalised patients [5]. Recent data from Malaysia, a middle-income country in Southeast Asia, has shown a 39% rise in the absolute number of incident HF hospitalisations from 2007 to 2016 (Su Miin Ong, MSc, unpublished data, 2020). This highlights an urgent need to fill the existing gaps on temporal trends for clinical outcomes, specifically for Asia [4]. Accordingly, our objective was to estimate the short- and medium-term mortality and readmission outcomes after an incident hospitalisation for HF. We then described the temporal variation in these outcomes by sex and ethnicity in this ethnically diverse country.

## Methods

### Data sources

We used hospital discharge data from the Health Informatics Centre, Ministry of Health, Malaysia from January 1, 2007 to December 31, 2016. Briefly, the Hospital Discharge Register represents a module within a centralised database known as the Patient Management Information System (*Sistem Maklumat Rawatan Pesakit*). It has been in operation since 1999 with the compilation of aggregated data on inpatient admissions. Then, granular patient records for Ministry of Health (MOH) hospitals became available by gradual increase in hospital participation over the years. Based on data from the Health Informatics Centre, inpatient admissions to public (MOH) hospitals make up to about 83% of HF hospitalisations in the country between 2008 and 2016, with the rest being hospitalisations in private, university and Ministry of Defence hospitals.

We included only data for MOH hospitals to maintain uniformity as data from other hospital types were available for only a later portion of the study. Mortality outcomes were determined via linkage with the National Mortality Database from the National Registration Department and Department of Statistics by matching on the national identification number or passport number for foreign nationalities in combination with date of birth and sex [6]. Overall, death registration in Malaysia has a coverage of more than 90%; there is complete registration in West Malaysia but under-reporting remains in East Malaysia (comprising the states of Sabah and Sarawak) [6,7,8]. Deaths in hospitals were medically-certified by attending physicians or coroners. On the other hand, deaths in the community were certified by informants such as the policemen and medical assistants and these were considered non-medically certified [9,10]. For the reporting of all-cause mortality, we considered all deaths, regardless of whether they were certified by medical personnel whereas for the calculation of cause-specific deaths, only those which were medically-certified were analysed.

### Study population

Patients aged 20 years and above, who were admitted to a MOH hospital for an incident HF hospitalisation were included. A discharge diagnosis of HF was defined by International Classification for Diseases version 10 (ICD-10) code I50 while a case was considered an incident hospitalisation if the patient had no admission for HF within the previous two years [11,12].

### Study outcomes and definitions

The main outcomes of this study were trends on 30-day readmission, in-hospital and 30-day mortality rates and lastly, mortality at one-year. In addition, we compared the crude and adjusted differences in these trends by sex and ethnicity. Lastly, we tabulated ICD-10 coded causes of death and readmission.

Deaths from any cause up to one year from date of HF admission were reported for years 2007 to 2016 while readmission data were only available till 2015. A readmission was defined as an admission for any cause within 30 days after discharge from an index HF hospitalisation while in-hospital mortality was defined as death which occurred during the index hospital admission. Information on ICD-10 coded causes of death was available only for seven years, 2007 till 2013. To examine temporal changes in HF admission criteria, we estimated trend changes in the average number of admissions per patient within one year from the index admission.

Information on age at incident HF hospitalisation, sex and ethnicity were available within the Hospital Discharge Register. In 2016, the Malaysian population is comprised of three major ethnic groups, in which there were 68.6% *Bumiputera* (made up of mainly Malays and Indigenous groups), 23.4% Chinese, 7.0% Indians and 1.0% other ethnicities [13]. In the medical records, ethnicity was self-reported and categorised into four groups as follows: (i) Malay, (ii) Chinese, (iii) Indian and (iv) Others which include Peninsular Malaysia Indigenous groups, Sabah and Sarawak Indigenous groups such as Bajau, Kadazan, Murut, Melanau, Kedayan, Iban, Bidayuh as well as non-Malaysian nationalities [14]. Because we anticipate a majority of Others to come from East Malaysia (consisting of two states, Sabah and Sarawak), which is known to have lower hospital densities and healthcare staff per population, and more remote communities than West Malaysia, we sought to determine the relative percentages of ethnicities by these two geographical regions [15].

### Ethical considerations

Ethics approval was obtained from the Medical Research and Ethics Committee, MOH (NMRR-19-1108-47994). A waiver of informed consent was granted as the analyses was done using observational data from routine clinical care. All data linkages between hospital discharge data and death records were conducted within the data environment of the Health Informatics Centre, MOH and only de-identified aggregated forms of the data were exported.

### Statistical methods

We reported crude readmission and mortality rates stratified by age, sex and ethnicity. For trends analyses, we standardised the outcome measures to the 2016 Malaysian population. Multivariable Poisson models was used to quantify the independent effect of age, sex, ethnicity and admission year on the study outcomes. An interaction term between admission year and ethnicity was used to determine if trend changes differed between ethnicities. There was evidence of overdispersion in the data, so quasi-Poisson models were used. A linear model was used to demonstrate statistical significance in the change in number of admissions per patient with time. Only the ethnicity variable had missing data for 0.3% observations and since this was a negligible percentage, they were excluded from the regression analysis. To assess robustness to changes in the definition of incident HF hospitalisation, we compared the estimates from one-year and three-year lookback periods with the main analyses. Statistical significance was set at 0.05. We used the R statistical software version 3.6.1 for all analyses [16].

## Results

### Patient characteristics, mortality and readmission rates

Between 2007 and 2016, there were 105 399 incident admissions for HF. The patients had a mean age of 64.1 (standard deviation (SD) 13.3) (***Table 1***). Fifty-six percent were men and 61% were Malays. Almost all of the Malay (97.8%), Chinese (87.8%) and Indian (99.6%) patients lived in West Malaysia while more than three quarters of Others (78.6%) live in the states of Sabah and Sarawak in East Malaysia.

**Table 1:** Patient characteristics and clinical outcomes for incident hospitalisations for heart failure.


YEAR	2007–2008		2009–2010		2011–2012		2013–2014		2015–2016	
				
	n	%	n	%	n	%	n	%	n	%

**Age**												

Mean (SD)	64.6	13.1	64.3	13.1	64.0	13.3	63.9	13.4	63.7	13.4

**Age group**												

20–<25	99	0.5	113	0.5	92	0.5	108	0.5	118	0.5

25–<30	147	0.8	171	0.8	184	0.9	186	0.9	214	0.9

30–<35	219	1.2	266	1.3	235	1.2	299	1.4	367	1.5

35–<40	328	1.7	358	1.7	384	2.0	428	2.0	603	2.4

40–<45	575	3.0	707	3.4	641	3.3	740	3.5	920	3.7

45–<50	1123	5.9	1252	5.9	1211	6.2	1244	5.9	1530	6.2

50–<55	1815	9.6	1948	9.2	1898	9.7	1956	9.3	2264	9.1

55–<60	2228	11.8	2599	12.3	2516	12.9	2762	13.1	3177	12.8

60–<65	2603	13.7	3036	14.4	2803	14.4	3190	15.2	3744	15.1

65–<70	2733	14.4	2931	13.9	2666	13.7	2925	13.9	3657	14.7

70–<75	2869	15.2	3184	15.1	2654	13.6	2615	12.4	3046	12.3

75–<80	2106	11.1	2211	10.5	2144	11.0	2391	11.4	2612	10.5

80–<85	1284	6.8	1420	6.7	1276	6.5	1332	6.3	1628	6.6

85+	803	4.2	868	4.1	799	4.1	879	4.2	965	3.9

**Sex**												

Male	10459	55.2	11743	55.7	10808	55.4	11774	55.9	14082	56.7

Female	8473	44.8	9321	44.3	8695	44.6	9281	44.1	10763	43.3

**Geographical region**												

West Malaysia	16615	87.8	18891	89.7	17459	89.5	18541	88.1	21989	88.5

East Malaysia	2317	12.2	2173	10.3	2044	10.5	2514	11.9	2856	11.5

**Ethnicity**												

Malay	11233	59.3	12732	60.4	12102	62.1	12837	61.0	15242	61.3

Chinese	3522	18.6	3968	18.8	3471	17.8	3764	17.9	4433	17.8

Indian	2182	11.5	2355	11.2	2123	10.9	2284	10.8	2585	10.4

Others	1922	10.2	1793	8.5	1798	9.2	2156	10.2	2585	10.4

Missing	73	0.4	216	1.0	9	0.05	14	0.1	0	0

**Length of Stay**												

Median (IQR)	3	2–5	3	2–5	3	2–5	3	2–5	3	2–5

Mean (SD)	4.7	12.2	4.5	14.4	4.3	7.0	4.2	7.0	4.1	5.0

**Number of admissions per year**												

Median (IQR)	1	0–2	1	0–2	1	0–2	1	0–2	1	0–2

Mean (SD)	1.3	2.2	1.4	2.3	1.3	2.1	1.5	2.3	1.6	2.5

**Mortality rate**												

In-hospital	1309	6.9	1297	6.2	1177	6.0	859	4.1	918	3.7

30-days	2482	13.1	2526	12.0	2285	11.7	2076	9.9	2406	9.7

1-year	6531	34.5	6945	33.0	6299	32.3	6965	33.1	8163	32.9

**Readmission rate**												

30-days	3144	16.6	3795	18.0	3387	17.4	4039	19.2	2421	19.6^†^

**Total**	**18932**		**21064**		**19503**		**21055**		**24845**	


^†^ Data only available for 2015 and its denominator is 12373.

A total of 16 786 (18.1%; 95% confidence interval (CI) 17.8–18.3%) patients had a readmission within 30 days of discharge. Mortality rates of hospitalised patients were 5.3% (95% CI 5.1–5.4%) during inpatient stay, 11.2% (95% CI 11.0–11.4%) within 30 days and 33.1% (95% CI 32.9–33.4%) within a year. The median length of hospital stay (LOS) was three days (interquartile range (IQR) 2–5). No association was found between LOS and in-hospital mortality. (Incidence rate ratio = 1.00, p-value = 0.529, adjusted for age, sex, ethnicity and time trend) Absolute risks for readmission and mortality by age, sex and ethnicity are displayed in Supplementary Table 1.

Thirty-day readmission rates were higher in men (19.4%) than women (16.4%). Although there were no apparent differences for inpatient mortality by sex (adjusted p = 0.340), mortality rates at 30 days and at one year were greater in men than women (11.4% vs 10.9% and 34.7% vs 31.2%, both adjusted p < 0.001) (***Table 2***). Age at index hospitalisation was a significant determinant of both short- and medium-term (one year) mortality. Patients who were on the extreme ends of the age spectrum, i.e., those aged 20–<25 years and 85 years and older had 2.4- and 1.8-fold increased risk of in-hospital death compared to those who were between 60 and 65 years. For patients who survived past 30 days, the risk of mortality within a year increased gradually from the 30–<35 years age band (adjusted risk ratio 0.85, 95% CI 0.77–0.94) to the oldest age band of 85 years and above (adjusted risk ratio 1.58, 95%CI 1.49–1.67) compared to the reference age category (60–<65 years).

**Table 2:** Multivariable Poisson regression analysis for readmission and mortality rates during hospital stay, 30 days and 1 year.


	READMISSION			MORTALITY								
	
	30-DAY			IN-HOSPITAL			30-DAY			1-YEAR		
			
	RATE RATIO (95% CI)		p-VALUE	RATE RATIO (95% CI)		p-VALUE	RATE RATIO (95% CI)		p-VALUE	RATE RATIO (95% CI)		p-VALUE

**Time**	1.02	(1.01–1.03)	***	0.93	(0.92–0.94)	***	0.96	(0.96–0.97)	***	1.00	(0.99–1.00)	

**Sex (ref = Women)**												

Men	1.16	(1.13–1.20)	***	1.02	(0.97–1.08)		1.11	(1.07–1.15)	***	1.17	(1.15–1.19)	***

**Ethnicity (ref = Malay)**												

Chinese	1.12	(1.08–1.16)	***	1.21	(1.12–1.30)	**	1.08	(1.03–1.13)	**	0.95	(0.93–0.98)	***

Indian	1.12	(1.07–1.17)	**	0.82	(0.74–0.91)	**	0.80	(0.75–0.85)	***	0.87	(0.84–0.90)	***

Others	0.81	(0.77–0.86)	***	1.91	(1.77–2.07)	***	1.30	(1.23–1.37)	***	0.96	(0.93–0.99)	*

**Age (ref = 60–<65)**												

20–<25	1.14	(0.94–1.37)		2.49	(1.88–3.22)	***	1.73	(1.40–2.11)	***	0.98	(0.85–1.12)	

25–<30	1.01	(0.86–1.17)		2.62	(2.12–3.21)	***	1.75	(1.48–2.04)	***	0.99	(0.89–1.10)	

30–<35	0.83	(0.72–0.95)	**	2	(1.64–2.43)	***	1.39	(1.20–1.60)	***	0.86	(0.78–0.94)	**

35–<40	0.98	(0.88–1.08)		1.25	(1.01–1.53)	*	1.09	(0.95–1.25)		0.83	(0.76–0.89)	***

40–<45	0.95	(0.87–1.03)		1.15	(0.96–1.36)		0.98	(0.88–1.10)		0.83	(0.78–0.88)	***

45–<50	0.94	(0.88–1.01)		0.88	(0.75–1.02)		0.84	(0.76–0.93)	**	0.82	(0.78–0.86)	***

50–<55	0.99	(0.93–1.05)		0.85	(0.74–0.96)	*	0.82	(0.75–0.89)	***	0.87	(0.83–0.91)	***

55–<60	0.98	(0.93–1.03)		0.93	(0.83–1.05)		0.90	(0.83–0.97)	**	0.93	(0.89–0.96)	***

65–<70	0.97	(0.92–1.02)		1.07	(0.96–1.20)		1.08	(1.01–1.16)	*	1.05	(1.02–1.09)	**

70–<75	0.91	(0.87–0.96)	**	1.14	(1.02–1.27)	*	1.23	(1.15–1.31)	***	1.15	(1.11–1.20)	***

75–<80	0.86	(0.81–0.91)	***	1.26	(1.12–1.41)	***	1.34	(1.25–1.44)	***	1.24	(1.19–1.29)	***

80–<85	0.84	(0.79–0.90)	***	1.53	(1.36–1.73)	***	1.59	(1.47–1.71)	***	1.37	(1.31–1.43)	***

85+	0.75	(0.69–0.81)	***	1.84	(1.61–2.10)	***	1.94	(1.79–2.11)	***	1.59	(1.51–1.66)	***

**Intercept**	0.16	(0.15–0.17)	***	0.06	(0.06–0.07)	***	0.11	(0.10–0.12)	***	0.3	(0.29–0.31)	***


* p < 0.05, ** p < 0.01, *** p < 0.001.

By ethnicity, 30-day readmission rates were highest in Indians (19.9%), followed by Chinese (19.5%), Malay (17.9%) and Others (14.5%). For short-term mortality, Others presented with the poorest outcomes: 9.4% for in-hospital mortality and 14.4% for 30-day mortality compared to 4.6% and 10.7% in Malays, the largest ethnic group in Malaysia. This translates to a 1.9-and 1.3-fold increase in risk for mortality relative to Malays when adjusted for age, sex and calendar year. Indian patients, on the other hand, had lower inpatient (3.8%), 30-day (8.4%) and one-year (28.6%) mortality rate than Malays. All ethnic differences in outcome measures remained when estimates were adjusted for age, sex and calendar year.

### Trends for readmission and mortality

**Figure 1:**
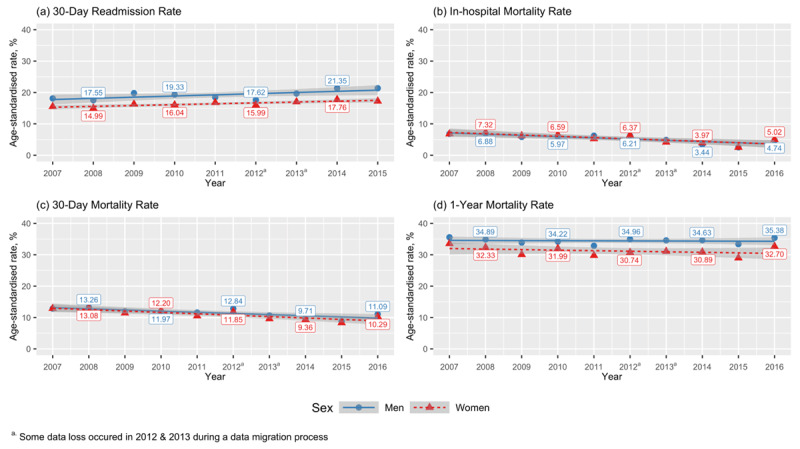
Trends for **(a)** 30-day readmission, **(b)** in-hospital, **(c)** 30-day and **(d)** one-year all-cause mortality rates by men and women.

**Figure 2:**
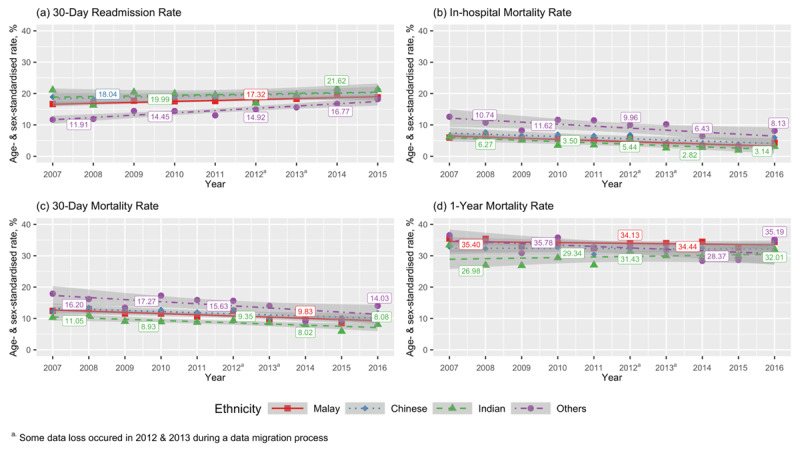
Trends for **(a)** 30-day readmission, **(b)** in-hospital, **(c)** 30-day and **(d)** one-year all-cause mortality rates by ethnicity.

A 17.8% increase in overall readmissions from 16.6% in 2007–2008 to 19.6% in 2015 was observed and this trend remained significant at +2% per calendar year after adjusting for age, sex and ethnicity (p trend < 0.001). ***Figure 1(a)*** shows that age-standardised trends for 30-day readmission in men were proportionally higher than the rate for women. Although Others had the lowest readmission rates in 2007, its rise with time was the largest compared to the other ethnic groups (***Figure 2 (a)***). The mean number of hospitalisations per patient within a year has risen slightly from 1.3 in 2007 to 1.6 in 2015 and a modest increase of 0.04 per calendar year was still evident after accounting for age, sex and ethnicity (p trend < 0.001).

Overall, in-hospital mortality rates nearly halved from 6.9% (95% CI 6.6–7.3) in 2007 to 3.7% (95% CI 3.5–3.9) in 2016 with similar trends for men and women (***Figure 1(b)***). This declining trend remained after adjustment for age, sex and ethnicity (average –7% per year; p trend < 0.001). Improvements in mortality were also evident within 30 days of hospitalisation, seen as a 26% decline from 13.1% (95% CI 12.6–13.6%) in 2007 to 9.7% (95% CI 9.3–10.1%) in 2016. Men had consistently higher 30-day mortality rates than women during the study period (***Figure 1(c)***). Upon full-model adjustment, the average improvement in 30-day mortality was 4% per calendar year (p trend < 0.001). By contrast, all-cause mortality in one year remained unchanged throughout the study period (p trend = 0.113) with men having almost uniformly higher rates than women (***Figure 1(d)***).

Despite poorer overall outcomes, Others showed the most pronounced improvements in short-term mortality over time compared to other groups (***Figure 2(b)*** and ***(c)***). The rates for 30-day mortality between Others and Malays narrowed from 6.2% in 2007 to 3.5% in absolute rate difference in 2016 but this difference was not significant after full model adjustment (p interaction = 0.091; Supplementary Table 2). With respect to 1-year mortality, only Others showed significant average decline by 1% per year (p interaction = 0.021) relative to Malays (***Figure 2(d)***, Supplementary Table2).

### Cause of readmission and death

**Figure 3:**
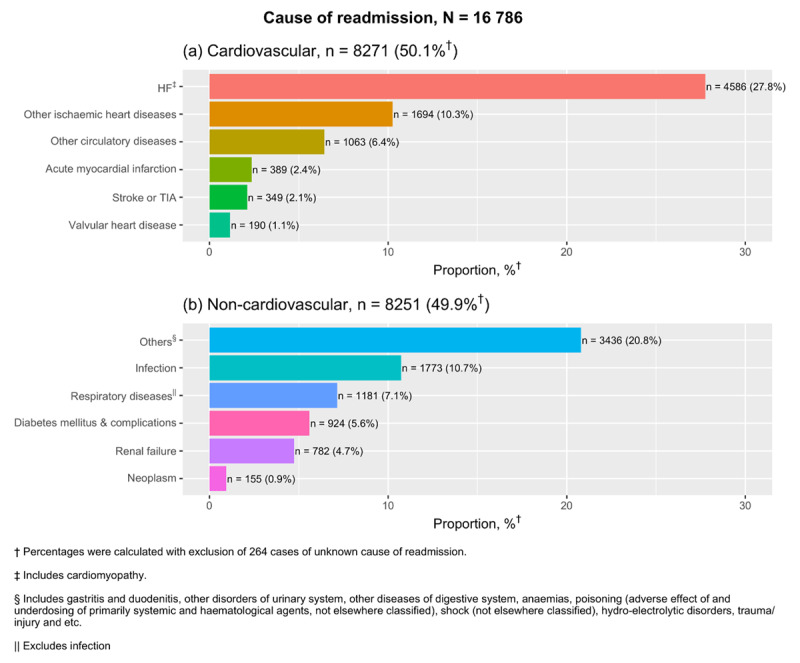
Causes of readmission in 30 days after index hospital admission from 2007–2015.

**Figure 4:**
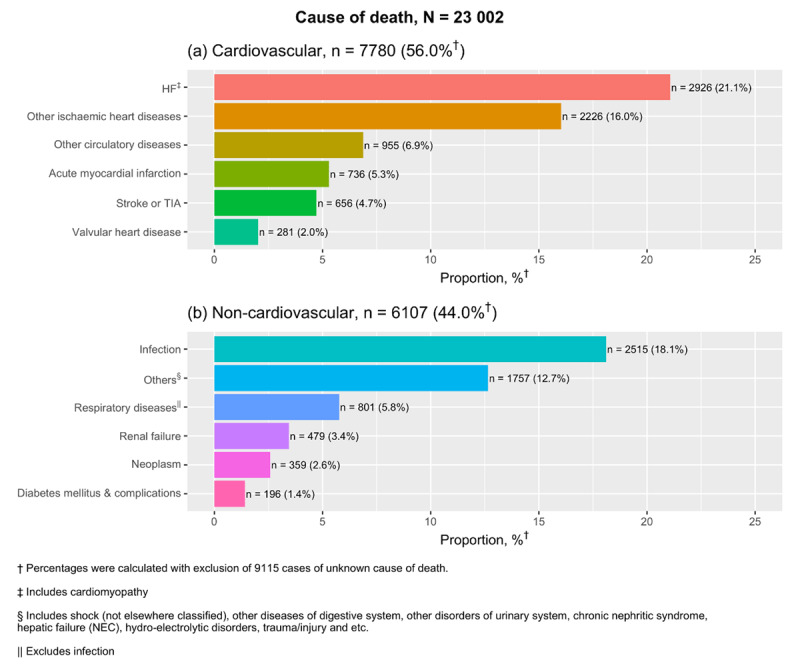
Causes of death for patients who died within one year after index admission from 2007–2013.

Cardiovascular causes accounted for half of all 30-day readmissions (50.1%) (***Figure 3***) with HF specifically accounting for 27.8%. Analysis on all medically-certified deaths found 56% of patients died of cardiovascular causes within a year from index hospitalisation, with the leading cause being HF (21.1%) (***Figure 4***). Cardiovascular mortality rates have been decreasing by two percent per calendar year, with adjusted p trend = 0.001 (Supplementary table 3). Despite this decline, a corresponding increase in non-cardiovascular mortality with time resulted in the unchanged overall rates for one-year mortality and a look into the specific causes between two calendar year periods for 2007–2008 and 2012–2013 showed that the contribution of infections as a cause of non-cardiovascular deaths has been increasing (Supplementary Table 4).

When the definition of incident HF hospitalisation was increased from a two-year to three -year lookback period, the percentage of reduction in false positives were between 1.7 to 3% annually. Nevertheless, the magnitude of outcome rates, trends and statistical significance of regression estimates were similar to the main results, for both the one-year and three-year definitions of incident hospitalisations (Supplementary Tables 5 and 6).

## Discussion

We present here contemporary crude and age-standardised estimates for mortality and readmission rates among hospitalised HF patients in a middle-income Asian country. Using linked population data, this study has revealed improving trends in short-term mortality following an incident hospitalisation for HF in Malaysia. However, mortality at one year has remained constant while readmissions within thirty days rose steadily during the observation period. We have noted distinct differences in patient outcomes by sex and ethnicity. First, readmission rates were consistently higher in men compared to women and in Chinese and Indians compared to Malay patients. Second, overall short-term mortality outcomes were poorest in Others compared to all other ethnicities while men had slightly worse outcomes than women for 30-day mortality. Third, improvements in survival after HF hospitalisation varied by ethnic groups with Others showing the steepest decline in one-year mortality.

In-hospital mortality after incident HF hospitalisation in Malaysia was higher compared to the 4.1% reported from a HF registry in China [17]. Nevertheless, it is necessary to take into consideration that being enrolled in a registry is associated with better outcomes [18]. Next, we observed a seven percent decline for in-hospital mortality for both men and women. This is in contrast to a rise in Brazil, from 8.3% to 10.8% between 2008 and 2017 [19]. Similar to inpatient mortality, the rates of mortality within 30 days was also decreasing, albeit to a smaller extent. No direct comparisons were available for middle-income countries. However, the decline observed here were consistent to those observed in several high-income settings including Western Australia, the Netherlands, and Sweden [20,21,22,23]. Several explanations are possible. The observed decline in short-term mortality is partly a reflection of improving population health, as seen with life expectancy increases from 73.7 years in 2008 to 75 years in 2018 [24]. Other explanations include earlier identification of cases as a result of rising population health awareness, improved pre-hospital emergency services and an increase in the number of medical specialists in the past two decades [25,26]. MOH hospitals have experienced almost doubling of the number of emergency medicine specialists from 93 to 167 from 2010 to 2013 and a modest increase in the number of cardiologists from 47 to 53 within the same period [27]. There are no known changes in reimbursement practices or implementation of nationwide quality improvement programs during the study period, therefore we expect the coding of ICD-10 to remain consistent over this duration.

In keeping with earlier and recent studies from Western populations [28,29,30], we have also showed that men had higher risks of mortality and readmission than women after accounting for age, ethnicity and time trends. Poorer survival and higher readmission rates in men after HF hospitalisation may be explained by a predominant heart failure with reduced ejection fraction subtype among men and higher prevalence of macrovascular disease, myocardial ischemia and infarction, which underlie the aetiology of HF in this subgroup [31]. Unfortunately, the type of HF and aetiology were not available in the present data.

Amidst the overall improvements in short-term mortality, it is necessary to note that striking ethnic differences exists. Others had poorer outcomes than the rest of the population. This difference was apparent even in the presence of under-reporting of deaths in East Malaysia, from which the majority of Others reside. Hence, we expect the true estimates to be even higher than what was observed. The health status of this subgroup is known to be poorer compared to the general population and is characterised by lower socioeconomic status, shorter life expectancy, undernutrition, insulin resistance and lack of trust in modern medicine [32]. Moreover, accessibility to hospitals remains a challenge for some residents of the interior and remote parts of Sabah and Sarawak and it is also likely that this region has a higher prevalence of rheumatic heart disease which may contribute to poorer outcomes among those hospitalised for HF [33,34,35]. These findings highlight a need to improve access to healthcare and focus resources to narrow the disparities in short-term mortality particularly in Others. For short-term mortality trends, it is reassuring to observe that the largest disparity in mortality outcomes between Others and Malays have been narrowing. The remaining differences in mortality outcomes between ethnicity represents opportunities for health interventions.

About one in three patients hospitalised for HF in this study die within a year and this has remained fairly constant during the study period. Mortality at one year is almost 1.5-fold compared to the European Society of Cardiology Long Term Heart Failure registry, reflecting differences in income per capita, health systems and patient characteristics between Malaysia and the European and Mediterranean countries which participated in this registry [36]. Trend-wise, death due to cardiovascular disease has decreased and this could be the result of a higher use of disease-modifying therapy such as renin-aldosterone angiotensin system inhibitors and beta-blockers in recent years, as reported in several tertiary centres [37,38,39,40]. Numerous reports have shown that people of South Asian descent are predisposed to higher risk of ischaemic heart disease compared to the rest of the population [41]. Accordingly, our previous study had also found the highest incidence of HF hospitalisations amongst Indians in the Malaysian population. Interestingly though, when it comes to survival, be it short-term or at one-year, Indians had significantly better survival compared to other ethnic groups. This suggests a stronger influence of environmental and behavioural determinants over genetic influences in HF outcomes. Further investigation into use of HF medications and lifestyle factors by ethnic subgroups would hence be warranted.

Preventing decompensation is an important therapeutic goal after a diagnosis of HF. To our knowledge, there is no published data on 30-day readmission trends after HF hospitalisation among middle-income countries. The steady annual rise in 30-day readmissions that we have found were comparable to those reported in Spain, but in contrast to a two percent reduction among Veteran’s Affairs hospital admissions in the United States [42,43]. It is necessary though, to keep in mind that differences in healthcare financing and infrastructure exist between countries of middle- and high-income economies. Lowering admission threshold for HF is a potential reason for this observed rise in readmission rates in this study. Differential readmission rates by ethnicity were likely related to socioeconomic status and educational level. This is reflected as higher readmission rates among the Chinese and Indians who largely reside in urban locations, whereas greater access and logistical barriers to care exists among Others [44]. However, narrowing of the gap in readmission rates between Others and the population average suggests that physical access for Others to secondary care is improving over the last decade. The overall increasing trend in 30-day readmissions observed here deserve attention from researchers and policy makers alike because hospitalisations incur the greatest financial costs to HF health expenditures and about a quarter of these readmissions are preventable [45]. Standardised strategies to differentiate the severity of patients who present at the emergency department would be useful for risk stratifying them into those who require admission while the rest may be observed and treated on an outpatient basis. We know that half of these readmissions are due to non-cardiovascular causes; therefore, multi-faceted assessments which address all comorbidities can be incorporated into early care transition to outpatient clinics, nurse-led home visits and structured telephone monitoring, all of which have shown moderate effectiveness in reducing rehospitalisations [46,47].

In this study, we estimated the average prognosis after an index hospitalisation for HF using representative data from a large national database. These findings are generalisable to other middle-income countries with similar government-funded health systems and diverse ethnic composition. While most HF hospitalisation data for middle income countries in literature come from urban tertiary centres [4], we have presented here data across a range of hospitals within the public health sector in Malaysia. Unlike patient selection in disease registries, the inclusion of unselected cases of HF hospitalisations here allowed us to make reliable comparisons between sex and ethnic groups.

There were several limitations in this study. Complete data were available for only primary discharge diagnoses; therefore, the absolute number of HF hospitalisations was likely underestimated. Nevertheless, the trend data is unlikely to be affected by this underestimation as the selection criteria used was uniform across time points. We explored the use of secondary discharge diagnoses as proxy for underlying disease severity but found that data completeness was not consistent across time. Therefore, it is difficult to draw conclusions on the severity of patients who were hospitalised for HF. Information on HF subtypes (reduced, preserved and mid-range ejection fraction), medical history, treatments and device therapy were not available in the discharge database and thus, does not allow for correlation of these factors with HF outcomes. While this analysis encompassed an average of 83% of annual HF hospitalisations in Malaysia, it is necessary to point out that we have included only HF patients who were hospitalised in MoH hospitals. Thus, these results are not generalisable to patients treated in the community setting or private hospitals. The 30-day and one-year mortality estimates were slightly underestimated due to incomplete death registration in East Malaysia. Lastly, there were some data losses in 2012 and 2013 and imputations were not feasible in this situation because the exact number of missing records was not known.

To the best of our knowledge, this is one of the first studies to report national, age-standardised estimates for HF prognosis and trends for hospitalised HF patients in the South East Asian region. The declining trend and narrowing of ethnic differences for short-term mortality showed that these outcomes are amenable to targeted interventions. Moreover, the differential HF outcomes by sex and ethnicity seen here highlights the importance of incorporating these determinants into risk predictions models or when calculating likely accrual endpoints in the design of therapeutic studies.

## Conclusion

Gradual improvements in short-term mortality were seen across sex and ethnicities although relative differences between ethnic subgroups remain apparent. The steady rise in 30-day readmission post-discharge and stagnating 1-year mortality rate raises concern, signalling a need for pro-active efforts from policymakers, physicians and research in making HF as a priority disease area.

## Additional File

The additional file for this article can be found as follows:

10.5334/gh.1108.s1 Supplementary file. Rounds Evaluation Rubric in French and English.

## Figures and Tables

**Table:** 

HF	Heart failure
ICD	International Classification for Diseases
MOH	Ministry of Health

## References

[1] Lippi G, Sanchis-Gomar F. Global epidemiology and future trends of heart failure. AME Medical Journal. 2020; 5. DOI: 10.21037/amj.2020.03.03

[2] Braunwald E. The war against heart failure: the Lancet lecture. The Lancet. 2015; 385: 812–824. DOI: 10.1016/S0140-6736(14)61889-4 25467564

[3] Hemingway H, Croft P, Perel P, et al. Prognosis research strategy (PROGRESS) 1: A framework for researching clinical outcomes. BMJ. 2013; 346. DOI: 10.1136/bmj.e5595 PMC3565687 23386360

[4] Callender T, Woodward M, Roth G, et al. Heart failure care in low- and middle-income countries: A systematic review and meta-analysis. PLoS Med. 2014; 11: e1001699. DOI: 10.1371/journal.pmed.1001699 25117081 PMC4130667

[5] MacDonald MR, Tay WT, Teng T-HK, et al. Regional Variation of Mortality in Heart Failure With Reduced and Preserved Ejection Fraction Across Asia: Outcomes in the ASIAN-HF Registry. Journal of the American Heart Association. 2020; 9: e012199. DOI: 10.1161/JAHA.119.012199 31852421 PMC6988158

[6] Shamsuddin K, Lieberman E. Linking death reports from the Malaysian Family Life Survey-2 with birth and death certificates. Med J Malaysia. 1998; 53: 343–353. 10971976

[7] United Nations. Levels and trends of mortality since 1950. New York: United Nations; 1982.

[8] Rao C, Omar MA, Ganapathy SS, Tamin NSI. Strengthening Mortality Statistics for Health Programs in Malaysia: Lessons from the Field. Dr Sulaiman Al Habib Medical Journal. 2019; 1: 52–54. DOI: 10.2991/dsahmj.k.191214.003

[9] Omar A, Ganapathy SS, Anuar MFM, et al. Cause-specific mortality estimates for Malaysia in 2013: Results from a national sample verification study using medical record review and verbal autopsy. BMC Public Health. 2019; 19: 110. DOI: 10.1186/s12889-018-6384-7 30678685 PMC6345029

[10] Adnan TH, Bujang MA, Supramaniam P, et al. Trend Analysis of Medically Certified Death in Malaysia, 1995–2010. Health Informatics Journal. 2012; 6.

[11] Camplain R, Kucharska-Newton A, Cuthbertson CC, Wright JD, Alonso A, Heiss G. Misclassification of incident hospitalized and outpatient heart failure in administrative claims data: the Atherosclerosis Risk in Communities (ARIC) study. Pharmacoepidemiol Drug Saf. 2017; 26: 421–428. DOI: 10.1002/pds.4162 28120359 PMC5380482

[12] Griffiths RI, O’Malley CD, Herbert RJ, Danese MD. Misclassification of incident conditions using claims data: Impact of varying the period used to exclude pre-existing disease. BMC Med Res Methodol. 2013; 13: 32. DOI: 10.1186/1471-2288-13-32 23496890 PMC3602098

[13] Department of Statistics Malaysia. Current Population Estimates, Malaysia, 2014–2016. Putrajaya, Malaysia: Department of Statistics Malaysia; 2016.

[14] Ministry of Health Malaysia. National Health Data Dictionary, Health Informatics Standards. 2nd Edition. Kuala Lumpur: Ministry of Health Malaysia; 2007.

[15] Ministry of Health Malaysia, Harvard University TH Chan School of Public Health. Malaysia Health Systems Research: Contextual analysis of the Malaysian health system. Vol. 1. Putrajaya: Ministry of Health Malaysia; 2016.

[16] R Core Team. R: A language and environment for statistical computing. Vienna, Austria: R Foundation for Statistical. Computing; 2019.

[17] Zhang Y, Zhang J, Butler J, et al. Contemporary Epidemiology, Management, and Outcomes of Patients Hospitalized for Heart Failure in China: Results From the China Heart Failure (China-HF) Registry. J Card Fail. 2017; 23: 868–875. DOI: 10.1016/j.cardfail.2017.09.014 29029965

[18] Lund LH, Carrero J-J, Farahmand B, et al. Association between enrolment in a heart failure quality registry and subsequent mortality-a nationwide cohort study. Eur J Heart Fail. 2017; 19: 1107–1116. DOI: 10.1002/ejhf.762 28229520

[19] Fernandes ADF, Fernandes GC, Mazza MR, et al. A 10-year trend analysis of heart failure in the less developed brazil. Arquivos brasileiros de cardiologia. 2020; 114: 222–231. DOI: 10.36660/abc.20180321 32215488 PMC7077585

[20] Teng T-H K, Finn J, Hobbs M, Hung J. Heart failure: Incidence, case fatality, and hospitalization rates in Western Australia between 1990 and 2005. Circ Heart Fail. 2010; 3: 236–243. DOI: 10.1161/CIRCHEARTFAILURE.109.879239 20071655

[21] Mosterd A, Reitsma JB, Grobbee DE. Angiotensin converting enzyme inhibition and hospitalisation rates for heart failure in the Netherlands, 1980 to 1999: the end of an epidemic? Heart. 2002; 87: 75. DOI: 10.1136/heart.87.1.75 11751672 PMC1766961

[22] Schaufelberger M, Swedberg K, Köster M, Rosén M, Rosengren A. Decreasing one-year mortality and hospitalization rates for heart failure in Sweden; Data from the Swedish Hospital Discharge Registry 1988 to 2000. Eur Heart J. 2004; 25: 300–307. DOI: 10.1016/j.ehj.2003.12.012 14984918

[23] Jackson SL, Tong X, King RJ, Loustalot F, Hong Y, Ritchey MD. National Burden of Heart Failure Events in the United States, 2006 to 2014. Circ Heart Fail. 2018; 11: e004873. DOI: 10.1161/CIRCHEARTFAILURE.117.004873 30562099 PMC6424109

[24] Department of Statistics Malaysia. Press release: Abridged life tables, Malaysia 2016–2018. 2018.

[25] Hisamuddin NARN, Hamzah MS, Holliman CJ. Prehospital emergency medical services in Malaysia. J Emerg Med. 2007; 32: 415–421. DOI: 10.1016/j.jemermed.2006.08.021 17499697

[26] World Health Organization. Regional Office for Western Pacific. Malaysia health system review. Vol. 3. Manila: WHO Regional Office for the Western Pacific; 2012.

[27] Clinical Research Centre. National Healthcare Establishment and Workforce Statistics (Hospital) 2012–2013. Kuala Lumpur: Clinical Research Centre, Ministry of Health Malaysia; 2015.

[28] Ho KK, Anderson KM, Kannel WB, Grossman W, Levy D. Survival after the onset of congestive heart failure in Framingham Heart Study subjects. Circulation. 1993; 88: 107–115. DOI: 10.1161/01.CIR.88.1.107 8319323

[29] Vaartjes I, Hoes A, Reitsma J, et al. Age- and gender-specific risk of death after first hospitalization for heart failure. BMC Public Health. 2010; 10: 637. DOI: 10.1186/1471-2458-10-637 20969758 PMC3091563

[30] Taylor CJ, Ordóñez-Mena JM, Jones NR, et al. National trends in heart failure mortality in men and women, United Kingdom, 2000–2017. Eur J Heart Fail. 2021; 23: 3–12. DOI: 10.1002/ejhf.1996 PMC8287578 32892471

[31] Lam CSP, Arnott C, Beale AL, et al. Sex differences in heart failure. European Heart Journal. 2019; 40: 3859–3868c. DOI: 10.1093/eurheartj/ehz835 31800034

[32] Tuan Abdul Aziz TA, Teh LK, Md Idris MH, et al. Increased risks of cardiovascular diseases and insulin resistance among the Orang Asli in Peninsular Malaysia. BMC Public Health. 2016; 16. DOI: 10.1186/s12889-016-2848-9 27009064 PMC4806488

[33] Chew KS, Chan HC. Prehospital care in Malaysia: issues and challenges. International Paramedic Practice. 2011; 1: 47–51. DOI: 10.12968/ippr.2011.1.2.47

[34] Hussin N. Socio demographic profiles of rheumatic heart disease (RHD) patients in Sabah. International Journal of Public Health Research. 2016; 6: 736–740.

[35] Hussin N. Insights from a rheumatic heart disease registry in a tertiary centre in Sabah. International Journal of Public Health Research. 2017; 7: 757–764.

[36] Crespo-Leiro MG, Anker SD, Maggioni AP, et al. European Society of Cardiology Heart Failure Long-Term Registry (ESC-HF-LT): 1-year follow-up outcomes and differences across regions. Eur J Heart Fail. 2016; 18: 613–625. DOI: 10.1002/ejhf.566 27324686

[37] Chin SP, Sapari S, How SH, Sim KH. Managing congestive heart failure in a general hospital in Malaysia. Are we keeping pace with evidence? Med J Malaysia. 2006; 61: 278–283. 17240575

[38] Chong A-Y, Rajaratnam R, Hussein NR, Lip GYH. Heart failure in a multiethnic population in Kuala Lumpur, Malaysia. Eur J Heart Fail. 2003; 5: 569–574. DOI: 10.1016/S1388-9842(03)00013-8 12921820

[39] Reyes EB, Ha J-W, Firdaus I, et al. Heart failure across Asia: Same healthcare burden but differences in organization of care. International Journal of Cardiology. 2016; 223: 163–167. DOI: 10.1016/j.ijcard.2016.07.256 27541646

[40] Zainal Abidin HA, Isa MR, Mohd Arshad MK, et al. Guideline Adherence to Prescription in Heart Failure Population in North Kuala Lumpur Region. International Journal of Cardiology. 2017; 249: S38. DOI: 10.1016/j.ijcard.2017.09.135

[41] Chaturvedi N. Ethnic differences in cardiovascular disease. Heart. 2003; 89: 681–686. DOI: 10.1136/heart.89.6.681 12748237 PMC1767706

[42] Fernandez-Gasso L, Hernando-Arizaleta L, Palomar-Rodríguez JA, Abellán-Pérez MV, Pascual-Figal DA. Trends, causes and timing of 30-day readmissions after hospitalization for heart failure: 11-year population-based analysis with linked data. Int J Cardiol. 2017; 248: 246–251. DOI: 10.1016/j.ijcard.2017.07.094 28801153

[43] Parizo JT, Kohsaka S, Sandhu AT, Patel J, Heidenreich PA. Trends in Readmission and Mortality Rates Following Heart Failure Hospitalization in the Veterans Affairs Health Care System From 2007 to 2017. JAMA Cardiol. 2020; 5: 1042–1047. DOI: 10.1001/jamacardio.2020.2028 32936253 PMC7301300

[44] Mariapun J, Ng C-W, Hairi NN. The Gradual Shift of Overweight, Obesity, and Abdominal Obesity Towards the Poor in a Multi-ethnic Developing Country: Findings From the Malaysian National Health and Morbidity Surveys. J Epidemiol. 2018; 28: 279–286. DOI: 10.2188/jea.JE20170001 29657257 PMC5976871

[45] Ziaeian B, Fonarow GC. The Prevention of Hospital Readmissions in Heart Failure. Progress in Cardiovascular Diseases. 2016; 58: 379–385. DOI: 10.1016/j.pcad.2015.09.004 26432556 PMC4783289

[46] Feltner C, Jones CD, Cené CW, et al. Transitional care interventions to prevent readmissions for persons with heart failure: A systematic review and meta-analysis. Ann Intern Med. 2014; 160: 774–784. DOI: 10.7326/M14-0083 24862840

[47] Chan WX, Lin W, Wong RCC. Transitional Care to Reduce Heart Failure Readmission Rates in South East Asia. Card Fail Rev. 2016; 2: 85–89. DOI: 10.15420/cfr.2016:9:2 28785458 PMC5490879

