# Supplementary material for: Trends for Readmission and Mortality After Heart Failure Hospitalisation in Malaysia, 2007 to 2016
Source: Glob Heart. 2022 Mar 8;17(1):20. doi: 10.5334/gh.1108 (PMC8916062; doi:10.5334/gh.1108)
Supplement: Supplementary file. — Rounds Evaluation Rubric in French and English. [file gh-17-1-1108-s1.pdf]

Supplementary file

Supplementary table 1. Readmission and mortality risk by age, sex and ethnicity

	30-day readmission n=16753		Mortality In-hospital n=5537		Mortality 30-day n=11742		Mortality 1-year n= 34843	
	No. of events	%	No. of events	%	No. of events	%	No. of events	%
**Age**								
20-<25	98	21.2	65	12.5	92	17.6	161	30.8
25-<30	150	18.9	115	12.9	157	17.6	278	31.2
30-<35	188	15.9	132	9.6	192	14.0	375	27.3
35-<40	334	19.0	119	5.7	223	10.7	545	26.2
40-<45	569	18.4	188	5.3	346	9.7	934	26.3
45-<50	1017	18.3	251	4.0	521	8.2	1644	26.0
50-<55	1676	19.2	380	3.9	793	8.0	2710	27.5
55-<60	2231	19.1	550	4.2	1160	8.8	3893	29.4
60-<65	2614	19.4	685	4.5	1496	9.7	4837	31.5
65-<70	2435	18.7	730	4.9	1580	10.6	4916	33.0
70-<75	2236	17.5	765	5.3	1744	12.2	5166	36.0
75-<80	1672	16.4	669	5.8	1512	13.2	4380	38.3
80-<85	984	16.1	506	7.3	1093	15.8	2919	42.1
85+	549	14.3	382	8.9	833	19.3	2085	48.4
**Sex**								
Men	10009	19.4	3070	5.2	6706	11.4	20384	34.7
Women	6744	16.4	2467	5.3	5036	10.9	14459	31.2
**Ethnicity**								
Malay	10120	17.9	2982	4.6	6862	10.7	21573	33.6
Chinese	3307	19.5	1160	6.1	2442	12.7	6642	34.7
Indian	2035	19.9	434	3.8	963	8.4	3292	28.6
Others	1291	14.5	961	9.4	1475	14.4	3336	32.5
**Overall**								

Rate (95% CI)	18.1	17.8-18.3	5.3	5.1-5.4	11.2	11.0-11.4	33.2	32.9-33.4

Supplementary table ~~1~~2. Multivariable Poisson regression analysis for 30-day readmission, in-hospital, 30-day and 1-year mortality rates with interaction term between time and ethnicity to determine statistical significance of trend differences by ethnicity

	**30-days readmission**			**In-hospital mortality**			**30-days mortality**			**1-year mortality**		
	Rate ratio	*CI*	*p*	*Rate ratio*	*CI*	*p*	*Rate ratio*	*CI*	*p*	*Rate ratio*	*CI*	*p*
(Intercept)	0.16	0.15 – 0.17	**<0.001**	0.06	0.06 – 0.07	**<0.001**	0.11	0.10 – 0.12	**<0.001**	0.30	0.29 – 0.31	**<0.001**
Time	1.02	1.01 – 1.02	**<0.001**	0.93	0.91 – 0.94	**<0.001**	0.97	0.96 – 0.97	**<0.001**	1.00	0.99 – 1.00	0.231
Time: Chinese	1.00	0.98 – 1.01	0.578	1.01	0.99 – 1.04	0.349	1.00	0.99 – 1.02	0.858	1.00s	0.99 – 1.01	0.738
Time: Indian	1.00	0.98 – 1.02	0.924	0.98	0.94 – 1.01	0.195	0.99	0.97 – 1.02	0.504	1.01	1.00 – 1.03	**0.010**
Time: Others	1.03	1.01 – 1.05	**0.002**	1.00	0.98 – 1.03	0.920	0.98	0.97 – 1.00	0.091	0.99	0.98 – 1.00	**0.021**
Malay	*Reference*											
Chinese	1.14	1.05 – 1.24	**0.002**	1.14	0.98 – 1.31	0.089	1.07	0.97 – 1.18	0.155	0.96	0.91 – 1.01	0.156

Indian	1.11	1.01 – 1.23	**0.038**	0.93	0.75 – 1.14	0.474	0.83	0.73 – 0.96	**0.010**	0.80	0.74 – 0.86	**<0.001**
Others	0.68	0.60 – 0.77	**<0.001**	1.90	1.63 – 2.22	**<0.001**	1.42	1.26 – 1.59	**<0.001**	1.03	0.96 – 1.11	0.369
Female	*Reference*											
Male	1.16	1.13 – 1.19	**<0.001**	1.02	0.97 – 1.08	0.429	1.11	1.07 – 1.15	**<0.001**	1.17	1.15 – 1.19	**<0.001**
60-<65	*Reference*											
20-<25	1.14	0.94 – 1.37	0.167	2.49	1.88 – 3.22	**<0.001**	1.73	1.40 – 2.11	**<0.001**	0.98	0.85 – 1.12	0.759
25-<30	1.01	0.86 – 1.17	0.918	2.62	2.12 – 3.21	**<0.001**	1.75	1.48 – 2.04	**<0.001**	0.99	0.89 – 1.10	0.884
30-<35	0.83	0.72 – 0.95	**0.008**	2.00	1.64 – 2.43	**<0.001**	1.39	1.20 – 1.61	**<0.001**	0.86	0.78 – 0.94	**0.002**
35-<40	0.98	0.88 – 1.08	0.654	1.25	1.01 – 1.53	**0.034**	1.09	0.95 – 1.25	0.224	0.83	0.76 – 0.89	**<0.001**
40-<45	0.95	0.87 – 1.03	0.195	1.15	0.96 – 1.36	0.115	0.99	0.88 – 1.10	0.796	0.83	0.78 – 0.88	**<0.001**
45-<50	0.94	0.88 – 1.01	0.078	0.88	0.75 – 1.02	0.089	0.84	0.76 – 0.93	**0.001**	0.82	0.78 – 0.87	**<0.001**
50-<55	0.99	0.93 – 1.05	0.696	0.84	0.74 – 0.96	**0.013**	0.82	0.75 – 0.89	**<0.001**	0.87	0.83 – 0.91	**<0.001**
55-<60	0.98	0.93 – 1.03	0.463	0.93	0.83 – 1.05	0.253	0.90	0.83 – 0.97	**0.005**	0.93	0.90 – 0.96	**<0.001**

65-<70	0.97	0.92 – 1.02	0.215	1.07	0.96 – 1.20	0.220	1.08	1.01 – 1.15	**0.033**	1.05	1.01 – 1.09	**0.006**
70-<75	0.91	0.87 – 0.96	**0.001**	1.14	1.02 – 1.27	**0.020**	1.23	1.15 – 1.31	**<0.001**	1.15	1.12 – 1.20	**<0.001**
75-<80	0.86	0.81 – 0.91	**<0.001**	1.25	1.12 – 1.40	**<0.001**	1.34	1.25 – 1.44	**<0.001**	1.24	1.19 – 1.28	**<0.001**
80-<85	0.84	0.79 – 0.90	**<0.001**	1.53	1.35 – 1.73	**<0.001**	1.59	1.47 – 1.71	**<0.001**	1.37	1.31 – 1.43	**<0.001**
85+	0.75	0.68 – 0.81	**<0.001**	1.84	1.61 – 2.10	**<0.001**	1.94	1.79 – 2.11	**<0.001**	1.59	1.52 – 1.66	**<0.001**
Observations	999			1111			1111			1111		
Deviance	894.324			1299.643			1114.947			890.756		

Supplementary table 32. Poisson model for cardiovascular and non-cardiovascular death at one year after index HF admission

	Cardiovascular death			Non-cardiovascular death		
Predictors	*Rate Ratio*	*CI*	*p*	*Rate Ratio*	*CI*	*p*
(Intercept)	0.10	0.09 – 0.11	**<0.001**	0.08	0.08 – 0.09	**<0.001**
Time	0.98	0.97 – 0.99	**0.001**	1.02	1.01 – 1.03	**0.003**
Female	*Reference*					
Male	1.21	1.16 – 1.26	**<0.001**	1.09	1.04 – 1.14	**0.001**
Malay	*Reference*					
Chinese	1.10	1.04 – 1.16	**0.001**	1.14	1.08 – 1.21	**<0.001**
Indian	1.07	1.00 – 1.14	0.063	1.03	0.95 – 1.11	0.496
Others	1.12	1.04 – 1.20	**0.002**	1.17	1.08 – 1.26	**<0.001**
60-<65	*Reference*					

20-<25	1.56	1.13 – 2.08	**0.005**	1.87	1.38 – 2.48	**<0.001**
25-<30	1.59	1.30 – 1.93	**<0.001**	1.72	1.37 – 2.13	**<0.001**
30-<35	1.15	0.94 – 1.39	0.161	1.47	1.21 – 1.78	**<0.001**
35-<40	1.14	0.97 – 1.34	0.109	1.20	1.00 – 1.43	**0.049**
40-<45	1.06	0.93 – 1.20	0.382	0.93	0.80 – 1.08	0.333
45-<50	0.92	0.82 – 1.02	0.110	0.83	0.74 – 0.94	**0.003**
50-<55	0.95	0.87 – 1.04	0.291	0.88	0.79 – 0.97	**0.011**
55-<60	1.00	0.92 – 1.08	0.905	0.93	0.85 – 1.02	0.146
65-<70	1.02	0.94 – 1.10	0.657	0.96	0.88 – 1.05	0.408
70-<75	1.10	1.01 – 1.18	**0.020**	0.98	0.90 – 1.07	0.672
75-<80	1.08	0.99 – 1.17	0.074	1.08	0.98 – 1.18	0.109
80-<85	1.07	0.97 – 1.18	0.162	1.12	1.01 – 1.24	**0.032**
85+	1.10	0.98 – 1.23	0.109	1.20	1.07 – 1.36	**0.003**

Observations	686	678
R^2 Nagelkerke	0.277	0.299

Supplementary table 43. Comparison on causes of death between 2007-2008 and 2012-2013

	2007-2008		2012-2013	
**Cause of death**	**n**	**%[†]**	**n**	**%[†]**
**Cardiovascular**	**2311**	**60%**	**1955**	**53.5%**
HF + cardiomyopathy	956	24.8%	722	19.8%
Other ischaemic heart diseases	693	18.0%	590	16.1%
Other diseases of the circulatory system	263	6.8%	189	5.2%
Acute myocardial infarction	179	4.6%	210	5.7%
Stroke	146	3.8%	171	4.7%
Valvular heart disease	74	1.9%	73	2.0%
**Non-cardiovascular**	**1541**	**40%**	**1699**	**46.5%**
Infection	519	13.5%	797	21.8%
Other[‡]	464	12.0%	488	13.4%
Renal failure	140	3.6%	107	2.9%
Respiratory disease	237	6.2%	189	5.2%
Neoplasm	108	2.8%	87	2.4%
Diabetes mellitus and complications	73	1.9%	31	0.8%
**Missing**	2679		2254	

†Percentages were calculated based on all known causes of death

‡ includes shock (not elsewhere classified), other diseases of digestive system, other disorders of urinary system, chronic nephritic syndrome, hepatic failure (NEC), hydro-electrolytic disorders, trauma and other causes

Supplementary table 54. Multivariable Poisson regression analysis for readmission and mortality rates using incident HF hospitalisation definition of no prior admission in the past 1 year

	**30-days readmission**			**In-hospital mortality**			**30-days mortality**			**1-year mortality**		
Predictors	*Incidence Rate Ratios*	*CI*	*p*	*Incidence Rate Ratios*	*CI*	*p*	*Incidence Rate Ratios*	*CI*	*p*	*Incidence Rate Ratios*	*CI*	*p*
(Intercept)	0.16	0.15 – 0.17	**<0.001**	0.06	0.05 – 0.06	**<0.001**	0.11	0.10 – 0.11	**<0.001**	0.30	0.29 – 0.31	**<0.001**
Time	1.02	1.01 – 1.03	**<0.001**	0.93	0.92 – 0.93	**<0.001**	0.96	0.96 – 0.97	**<0.001**	1.00	0.99 – 1.00	0.102
Female	*Reference*			*Reference*			*Reference*			*Reference*		
Male	1.16	1.13 – 1.20	**<0.001**	1.02	0.96 – 1.08	0.486	1.11	1.07 – 1.15	**<0.001**	1.17	1.15 – 1.19	**<0.001**
Malay	*Reference*			*Reference*			*Reference*			*Reference*		
Chinese	1.11	1.07 – 1.16	**<0.001**	1.21	1.12 – 1.30	**<0.001**	1.08	1.03 – 1.13	**0.001**	0.96	0.93 – 0.98	**<0.001**
Indian	1.10	1.05 – 1.15	**<0.001**	0.82	0.73 – 0.91	**<0.001**	0.81	0.76 – 0.86	**<0.001**	0.88	0.85 – 0.91	**<0.001**
Others	0.82	0.78 – 0.87	**<0.001**	1.92	1.78 – 2.07	**<0.001**	1.30	1.23 – 1.37	**<0.001**	0.96	0.93 – 0.99	**0.007**
60-<65	*Reference*			*Reference*			*Reference*			*Reference*		
20-<25	1.13	0.92 – 1.36	0.228	2.54	1.92 – 3.28	**<0.001**	1.73	1.40 – 2.11	**<0.001**	0.98	0.85 – 1.12	0.770
25-<30	0.99	0.85 – 1.16	0.944	2.62	2.12 – 3.21	**<0.001**	1.74	1.48 – 2.04	**<0.001**	0.98	0.88 – 1.09	0.775
30-<35	0.83	0.72 – 0.96	**0.012**	2.03	1.66 – 2.46	**<0.001**	1.39	1.20 – 1.60	**<0.001**	0.86	0.78 – 0.94	**0.002**
35-<40	0.95	0.85 – 1.06	0.403	1.31	1.07 – 1.59	**0.008**	1.11	0.97 – 1.26	0.138	0.83	0.77 – 0.89	**<0.001**
40-<45	0.94	0.86 – 1.02	0.148	1.17	0.98 – 1.38	0.071	0.99	0.88 – 1.10	0.840	0.83	0.78 – 0.88	**<0.001**
45-<50	0.94	0.88 – 1.01	0.072	0.89	0.76 – 1.03	0.126	0.84	0.77 – 0.93	**<0.001**	0.83	0.79 – 0.87	**<0.001**

50-<55	1.00	0.94 – 1.06	0.996	0.87	0.76 – 0.99	**0.032**	0.83	0.76 – 0.90	**<0.001**	0.87	0.84 – 0.91	**<0.001**
55-<60	0.99	0.93 – 1.04	0.592	0.96	0.85 – 1.08	0.485	0.91	0.84 – 0.98	**0.010**	0.94	0.90 – 0.97	**0.001**
65-<70	0.98	0.93 – 1.03	0.467	1.09	0.98 – 1.21	0.130	1.08	1.01 – 1.16	**0.022**	1.05	1.02 – 1.09	**0.004**
70-<75	0.92	0.87 – 0.97	**0.003**	1.15	1.03 – 1.28	**0.013**	1.22	1.14 – 1.30	**<0.001**	1.15	1.11 – 1.19	**<0.001**
75-<80	0.87	0.82 – 0.92	**<0.001**	1.27	1.14 – 1.42	**<0.001**	1.34	1.25 – 1.44	**<0.001**	1.23	1.19 – 1.28	**<0.001**
80-<85	0.86	0.80 – 0.92	**<0.001**	1.54	1.36 – 1.73	**<0.001**	1.58	1.46 – 1.70	**<0.001**	1.36	1.31 – 1.42	**<0.001**
85+	0.75	0.68 – 0.82	**<0.001**	1.85	1.62 – 2.10	**<0.001**	1.93	1.78 – 2.10	**<0.001**	1.57	1.50 – 1.64	**<0.001**

Supplementary table 6~~5~~. Multivariable Poisson regression analysis for readmission and mortality rates using incident HF hospitalisation definition of no prior admission in the past 3 years

	**30-days readmission**			**In-hospital mortality**			**30-days mortality**			**1-year mortality**		
Predictors	*Incidence Rate Ratios*	*CI*	*p*	*Incidence Rate Ratios*	*CI*	*p*	*Incidence Rate Ratios*	*CI*	*p*	*Incidence Rate Ratios*	*CI*	*p*
(Intercept)	0.16	0.15 – 0.17	**<0.001**	0.06	0.05 – 0.07	**<0.001**	0.11	0.10 – 0.12	**<0.001**	0.29	0.28 – 0.30	**<0.001**
Time	1.02	1.01 – 1.03	**<0.001**	0.92	0.91 – 0.93	**<0.001**	0.96	0.96 – 0.97	**<0.001**	1.00	1.00 – 1.00	0.969
Female	*Reference*			*Reference*			*Reference*			*Reference*		
Male	1.16	1.13 – 1.20	**<0.001**	1.02	0.96 – 1.08	0.576	1.12	1.08 – 1.16	**<0.001**	1.17	1.15 – 1.19	**<0.001**

Malay	*Reference*			*Reference*			*Reference*			*Reference*		
Chinese	1.11	1.07 – 1.15	**<0.001**	1.23	1.14 – 1.33	**<0.001**	1.10	1.04 – 1.15	**<0.001**	0.96	0.93 – 0.98	**0.001**
Indian	1.10	1.05 – 1.15	**<0.001**	0.80	0.71 – 0.90	**<0.001**	0.80	0.74 – 0.85	**<0.001**	0.86	0.83 – 0.89	**<0.001**
Others	0.81	0.77 – 0.86	**<0.001**	1.91	1.76 – 2.07	**<0.001**	1.28	1.21 – 1.36	**<0.001**	0.94	0.91 – 0.98	**0.001**
60-<65	*Reference*			*Reference*			*Reference*			*Reference*		
20-<25	1.16	0.95 – 1.40	0.143	2.50	1.85 – 3.28	**<0.001**	1.78	1.42 – 2.19	**<0.001**	1.01	0.87 – 1.16	0.914
25-<30	1.00	0.85 – 1.17	0.967	2.72	2.18 – 3.37	**<0.001**	1.83	1.55 – 2.16	**<0.001**	1.03	0.92 – 1.15	0.612
30-<35	0.84	0.73 – 0.97	**0.020**	2.01	1.63 – 2.47	**<0.001**	1.39	1.19 – 1.62	**<0.001**	0.87	0.78 – 0.95	**0.004**
35-<40	0.97	0.87 – 1.08	0.607	1.22	0.97 – 1.51	0.083	1.08	0.93 – 1.25	0.299	0.82	0.75 – 0.89	**<0.001**
40-<45	0.94	0.86 – 1.03	0.166	1.14	0.95 – 1.36	0.166	0.98	0.86 – 1.10	0.697	0.83	0.77 – 0.88	**<0.001**
45-<50	0.95	0.88 – 1.02	0.126	0.88	0.75 – 1.03	0.121	0.84	0.76 – 0.93	**0.001**	0.83	0.79 – 0.87	**<0.001**
50-<55	0.99	0.94 – 1.06	0.839	0.83	0.72 – 0.96	**0.012**	0.81	0.74 – 0.89	**<0.001**	0.87	0.83 – 0.91	**<0.001**

55-<60	0.98	0.93 – 1.04	0.521	0.94	0.83 – 1.07	0.366	0.90	0.83 – 0.98	**0.013**	0.93	0.89 – 0.96	**<0.001**
65-<70	0.97	0.92 – 1.02	0.237	1.09	0.97 – 1.22	0.161	1.08	1.00 – 1.16	**0.042**	1.05	1.01 – 1.09	**0.017**
70-<75	0.92	0.87 – 0.97	**0.004**	1.14	1.01 – 1.28	**0.028**	1.23	1.14 – 1.32	**<0.001**	1.15	1.11 – 1.20	**<0.001**
75-<80	0.87	0.82 – 0.93	**<0.001**	1.23	1.09 – 1.39	**0.001**	1.32	1.22 – 1.42	**<0.001**	1.24	1.19 – 1.29	**<0.001**
80-<85	0.85	0.79 – 0.92	**<0.001**	1.55	1.36 – 1.77	**<0.001**	1.59	1.46 – 1.72	**<0.001**	1.37	1.31 – 1.43	**<0.001**
85+	0.75	0.68 – 0.82	**<0.001**	1.83	1.58 – 2.11	**<0.001**	1.93	1.76 – 2.11	**<0.001**	1.59	1.51 – 1.67	**<0.001**
